# 1175. Extended Infusion Beta-lactams in Pediatric Patients with Febrile Neutropenia

**DOI:** 10.1093/ofid/ofad500.1015

**Published:** 2023-11-27

**Authors:** Jerod Nagel, Karen Davidge, Madeleine King, Abdullah Al-Ajmi

**Affiliations:** Michigan Medicine, Ann Arbor, Michigan; University of Michigan Health, Ann Arbor, Michigan; University of Michigan Health, Ann Arbor, Michigan; Mayo Clinic, Byron, Minnesota

## Abstract

**Background:**

Febrile neutropenia (FN) is associated with a high risk for infections in patients receiving chemotherapy. Mortality estimates in pediatric patients with FN range from 0.4-3%. Extended infusion (EI) of beta-lactam antibiotics leads to improved pharmacodynamic parameters and is associated with enhanced antimicrobial activity. A recent study demonstrated significantly better overall treatment response in adult patients with documented infections and high-risk FN who received EI vs. standard infusion (SI) piperacillin-tazobactam. There are currently no studies evaluating clinical outcomes of EI beta-lactam use in pediatric patients with FN.

**Methods:**

This study was a single-center retrospective cohort study with a time frame between September 2016-September 2022. The primary objective was to assess the time to fever resolution in patients with FN who received EI vs. SI of beta-lactam antibiotics. Secondary objectives included the difference in 30-day mortality and hospital length of stay (LOS) between the EI and SI groups. This study included patients admitted to pediatric hematology, oncology, or BMT services at Michigan Medicine who were (1) diagnosed with FN during hospital admission or admitted for FN following chemotherapy and (2) treated with EI or SI cefepime, piperacillin-tazobactam, or meropenem for at least 48 hours.

**Results:**

A total of 87 patients were included in the study analysis (40 in the EI group and 47 in the SI group). Baseline characteristics were similar between the groups. There was no significant difference in the time to fever resolution for the cohort (48.5 vs 39.1 hours, p=0.373). There were numeric non-significant differences favoring EI for 30-day mortality (0% vs. 4%, p=0.496), and avoiding need to escalate antibiotic therapy due to treatment failure (7.5% vs. 17%, p=0.214).

**Conclusion:**

The small sample size limited the ability to detect differences in time to fever resolution between EI and SI beta-lactams. Although not statistically significant, secondary endpoints showed numerically lower mortality and lower rates of therapy escalation in the EI group.

**Disclosures:**

**Madeleine King, PharmD, BCOP**, Elsevier, Inc: Board Member

